# Changes of masseter muscle after mandible distraction osteogenesis in patients with Hemifacial microsomia: a retrospective study

**DOI:** 10.3389/fped.2024.1453270

**Published:** 2024-08-26

**Authors:** Wenqing Han, Byeong Seop Kim, Ziwei Zhang, Xiaojun Chen, Yingjie Yan, Li Lin, Yan Zhang, Gang Chai

**Affiliations:** Department of Plastic and Reconstructive Surgery, Shanghai Ninth People’s Hospital, Shanghai Jiao Tong University School of Medicine, Shanghai, China

**Keywords:** Hemifacial microsomia, masseter muscle, mandible distraction osteogenesis, CT, quantitative analysis

## Abstract

**Introduction:**

Mandible distraction osteogenesis (MDO) is widely used to reconstruct the mandible in patients with mild Hemifacial microsomia (HFM). However, the masseter's response to mandible distraction remains unclear.

**Methods:**

In this study, we analyze the effect of the surgical intervention on masseter muscle by a retrospective analysis. The procedure consisted of a five-day latent period, a three-week distraction period, and a six-month consolidation period. CT data were manually segmented and measured with Mimics software before surgery, within 3 months, and more than 1 year postoperatively. Masseter volume, masseter length, masseter width, and mandible ramus height were measured and analyzed using paired t-test, Pearson, and Spearman correlation analysis.

**Results:**

We included 21 patients with HFM who underwent mandible distraction osteogenesis from 2015 to 2020. The masseter volume on the affected side increased immediately after surgery from (6,505.33 ± 3,671.95) mm^3^ to (10,194.60 ± 5638.79) mm^3^, but decreased to (8,148.38 ± 3,472.57) mm^3^ at the second follow-up correlated to mandible ramus height (*r* = 0.395, *P* = 0.038). A similar trend was observed in changes in masseter length. Symmetry and width of masseter muscle had no longitudinal statistical significance.

**Discussion:**

Masseter muscle involvement benefits from MDO in the short term. To achieve long-term efficacy, more attention should be paid to muscle reconstruction.

## 1 Introduction

Hemifacial microsomia (HFM) is a congenital craniomaxillofacial disease characterized by mandibular hypoplasia and often involves masseter muscle morphology and function (1). Mandible distraction osteogenesis (MDO) is widely performed in patients with mild HFM (2). Masseter muscle interacts closely with the mandible through muscle-bone crosstalk (3) and responds to mandible surgery clinically (4). In addition, recurrence after MDO may be associated with ipsilateral masseter muscle capsule (5). Therefore, it is of great clinical significance to study the changes in masseter muscle after MDO.

According to existing animal studies, the muscle response after MDO depended to some extent on various distraction regimens, consolidation periods, and follow-up time. Castano FJ et al. reported increasing in Proliferating Cell Nuclear Antigen (PCNA) in 3 weeks after MDO in 16 Yucatan minipigs, suggesting that the presence of muscle proliferative response might contribute to the stability of distraction (6). Eighteen New Zealand rabbits showed atrophy, necrosis, and myophagocytosis in 3 months after MDO and disappeared in 6 months with adaptation (7). Distraction rate is also one of the influencing factors of muscle response. Gradual distraction showed regeneration in a natural pattern, while displacement at once made the equilibrium degenerate rather than regenerate (8, 9). Muscle response may also be related to the direction of distraction. The masseter muscle perpendicular to the vector of mandibular distraction showed atrophy according to enzyme and histomorphology, while the digastric muscle parallel to the vector adapted to MDO (10). Bone maturity should also be considered in the analysis. In studies of bone immaturity, the occlusal vertical dimension in the affected side increased with distraction, and a compensatory increase in volume occurred earlier than in the group with bone maturity (11). Chronic prolongation of neurally intact led to the addition of sarcomere in series in the bone immaturity group. At the same time, in skeletally mature animals, the same distraction regimen showed fibrosis and weakness for muscle prolongation, possibly due to denervation (12).

The response of soft tissue to bone distraction and its relationship with long-term stability remains unclear clinically. In Bilateral sagittal split osteotomy(BSSO), the tension of anterior-extension soft tissue is thought to be related to backward relapse (13). As for MDO, some studies reported the soft tissues were simultaneously lengthened, allowing effective regeneration (14, 15). On the contrary, K. Rafferty et al. reported masseter muscle was disrupted, resulting in reduced mechanical loading (16).

As we know, evaluation of masseter muscle and mandible based on CT is accurate and effective (17). In this retrospective study, we intended to descript the changes in masseter muscle (volume, width, length) and mandible after MDO and investigated the related factors.

## 2 Materials and methods

### 2.1 Participants

We retrospectively studied Pruzansky type II patients from 2015 to 2020. All patients received unilateral MDO and were followed up within 3 months and over 1 year after distractor removal. Patients with a history of masseter absence, other syndromes, cleft lip and palate, muscle disease, facial nerve involvement, facial trauma, other craniofacial surgical/physical treatments or other muscle treatment were excluded. Pre- and postoperative data included clinically standardized photographs and three-dimensional cranial CT. The CT was taken in a supine position with intercuspal position by the Light Speed 16 spiral CT (GE LightSpeed 16, Milwaukee, WI) with a thickness of less than 1 mm and saved in DICOM format with the 3D images reconstructed. Clinical examination included head and facial physical examination, facial nerve examination, and hearing examination. An expert panel performed OMENS + classification (18). This study has been approved by the Ethics Committee of Shanghai Ninth People's Hospital.

### 2.2 Protocol design and surgical treatment

In a previous study, we detailed our surgical design and procedures for maximum effective vertical extension (19). MDO was performed after adequate communication with the patient's guardians. Our procedure consisted of a latent period of five days, a distraction period of approximately three weeks, and a six-month consolidation period beginning after overcorrection. Then, the removal of screws and distractor was performed with the used incision. A CT scan was taken for postoperative evaluation within 3 months and over 1 year after the removal surgery.

### 2.3 Evaluation indexes

DICOM data was exported to Mimics 19.0 software (Materialise, Belgium) for manual annotation. The horizontal plane was marked with the unaffected infraorbital point and ear points, and the CT view was calibrated parallel to this plane. The masseter muscles were extracted by threshold setting, and the region of interest was manually delineated, assisted by the “region growing” tool. Examine edge segmentation in three views, and finally, the 3d reconstruction was performed. Reconfirm the muscle morphology clear, output volume measurement. The masseter muscle volume asymmetry was calculated using (UN_MV - AF_MV)/(UN_MV + AF_MV) * 100% where “UN_MV” denoted masseter muscle volume in the unaffected side and “AF_MV” for that of the affected side. The maximum masseter muscle width (MW) was selected perpendicular to the outer plate of the mandible on the maximum cross-sectional area. The masseter muscle (ML) length was measured from the apex of the mandibular notch to the anterior edge of the origin of the masseter muscles located at the zygomatic arch. The height of the mandible ramus (MRH) was recorded from the gonial to the uppermost point of the condyle.

### 2.4 Statistical methods

Continuous variables were presented as mean ± standard deviation. Paired *t*-tests were performed separately for preoperative and postoperative measurements. Pearson and Spearman correlation analyses were also performed to analyze masseter muscle change correlation factors.

## 3 Results

### 3.1 Baseline analysis

There were 21 patients (13 males and 8 females) included in the study, with age range from 3 to 12 years (mean age 6.19 ± 3.04). The OMENS + classification is shown in Table 1. According to mandibular involvement, the subjects were divided into M2a (8,38.1%); M2b(13,61.9%). In terms of soft tissue involvement, they were classified into S1(17,46.7%) and S2(4,13.3%). (Table 1) The preoperative (T0) mean AF_MV was (6,505.33 ± 3,671.95) mm^3^ and (UN_MV) was (12,288.67 ± 6,250.10) mm^3^. The masseter volume asymmetry was (30.60 ± 23.31)%. The bilateral differences of MV, ML, MW, and MRH were statistically significant. Correlation analyses of preoperative parameters showed AF_MV was related to age (r = 0.849, *P *< 0.001), M grade (r = 0.372, *P *= 0.048), O grade (r = 0.430, *P *= 0.026), UN_MV (r = 0.596, *P *= 0.002) and AF_MW (r = 0.577, *P *= 0.003) and AF_ML (r = 0.883, *P *< 0.001).

**Table 1:** Patient baseline data OMENS+.

		N, (%)
Gender	Male	13, (61.9%)
	Female	8, (38.1%)
Age (years)		6.14 ± 3.59
Side	Left	11, (52.4%)
	Right	10, (47.6%)
O	O0	14, (66.7%)
	O1	5, (23.8%)
	O2	2, (9.5%)
M	M2a	8, (38.1%)
	M2b	13, (61.9%)
E	E1	7, (33.3%)
	E2	7, (33.3%)
	E3	7, (33.3%)
N	N0	20, (100%)
S	S1	17, (81.0%)
	S2	4, (19.0%)

*OMENS + clasification: O, orbital; M mandible; E, ear; N, facial nerve; S, soft tissue.

### 3.2 Short-term following-up

At the short-term following up within 3 months (T1), AF_MV increased to (10,194.60 ± 5,638.79) mm^3^, and UN_MV was (13,981.61 ± 5,905.45) mm^3^. AF_ML and UN_ML were (31.18 ± 10.36) mm and (39.83 ± 9.02) mm. MV and ML were significantly increased compared to the baseline (*P *< 0.05). Muscle asymmetry improved by 4.16%. The increase in MRH on the affected side was correlated to the increase in AF_ML (r = 0.435, *p* = 0.031). No significant correlation was observed in MRH increase with the increase in AF_MW (r = 0.299, *p* = 0.189), AF_MV (r = 0.145, *p* = 0.531) or the asymmetry index (r = −0.295, *p* = 0.195).

### 3.3 Long-term following-up

**Figure 1:**
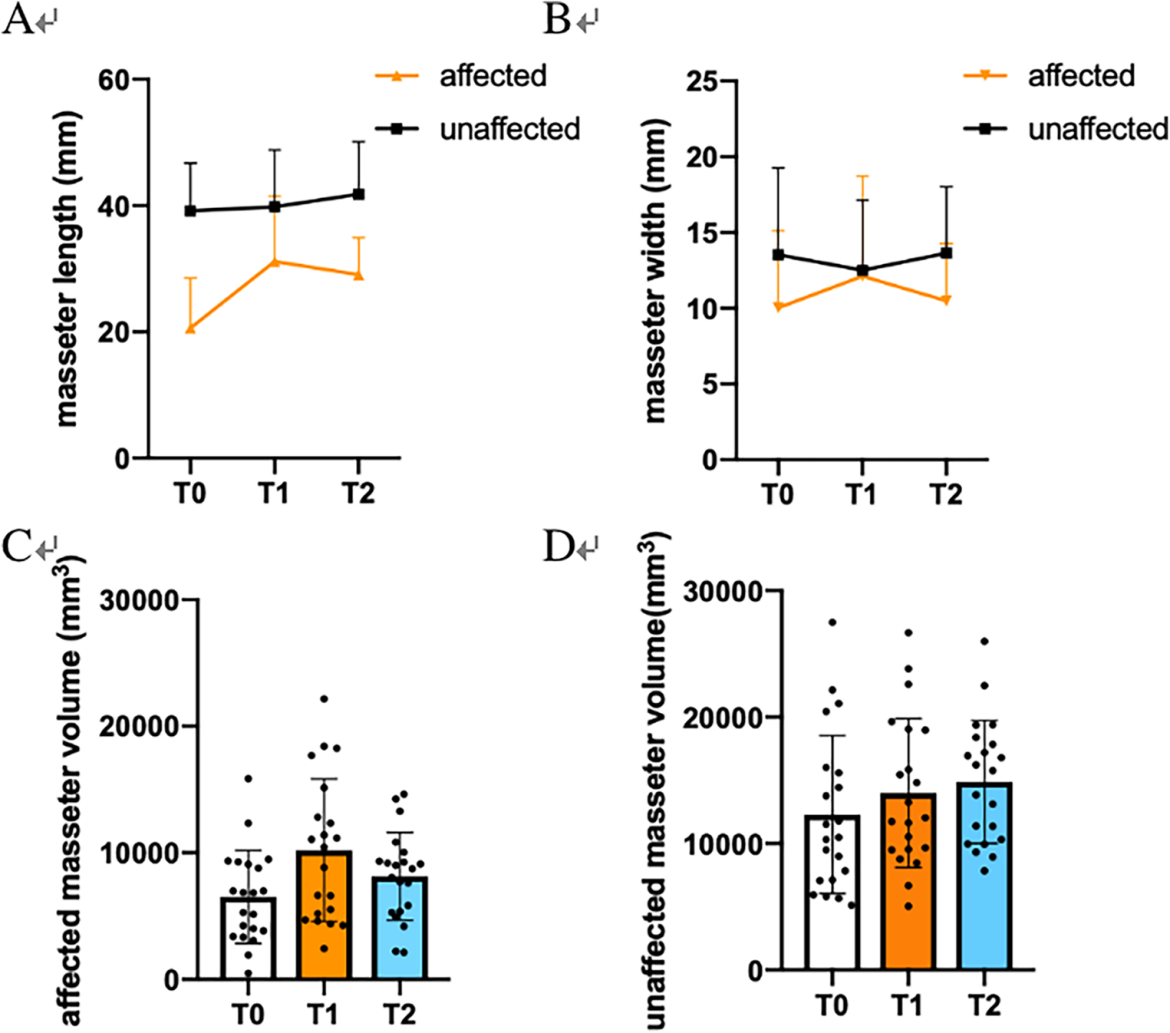
Statistical analysis of masseter changes before (T0), within 3 months (T1) and over 1 year (T2). **(A)** Masseter length (ML). **(B)** Masseter width (MW). **(C)** Affected masseter volume (MV). **(D)** Unaffected masseter volume.

As for over 1 year postoperation (T2), AF_MV decreased to (8,148.38 ± 3,472.57) mm^3^, and AF_ML was (29.05 ± 5.87) mm correlated to MRH (r = 0.395, *P *= 0.038). (Table 2) Both were lower than short-term follow-up. (Figure 1) No longitudinal statistical differences in asymmetry and width were observed throughout the study. Besides, no significance correlation was observed in MRH increase with the increase (decrease) of AF_MW, AF_MV or the asymmetry index.

**Table 2:** Masseter muscle and mandible measurements before (T0), within 3 months (T1), over 1 year (T2) after MDO.

	Masseter volume (mm^3^)		Asymmetry (%)	Masseter width (mm)		Masseter length (mm)		Mandible ramus height (mm)	
	AF_MV	UN_MV		AF_MW	UN_MW	AF_ML	UN_ML	AF_MRH	UN_MRH
T0	6,505.33 ± 3,671.95	12,288.66 ± 6,250.09	30.60 ± 23.31	10.04 ± 5.07	13.54 ± 5.72	20.60 ± 7.93	39.16 ± 7.57	29.05 ± 5.87	35.05 ± 7.87
T1	10,194.60 ± 5,638.79	13,981.61 ± 5,905.45	26.44 ± 16.70	12.11 ± 5.07	12.50 ± 4.63	31.18 ± 10.36	39.83 ± 9.02	33.83 ± 8.76	36.66 ± 7.36
T2	8,148.38 ± 3,472.57	14,871.73 ± 4,861.86	30.84 ± 15.50	10.49 ± 3.77	13.64 ± 4.36	29.05 ± 5.87	41.84 ± 8.29	31.92 ± 8.17	38.54 ± 8.10

*UN-, unaffected side; AF-, affected side; MV, masseter volume; MW, masseter width; ML, masseter length; MRH, masseter ramus length.

## 4 Discussion

Masseter muscle parameters are not only a manifestation of HFM involvement but also a factor in the remodeling process of the skeleton (3). However, postoperative masseter muscle changes remain controversial. Based on CT data, this study retrospectively analyzed the influence of masseter morphology after MDO. Our results show masseter volume in the affected side increased immediately after MDO and decreased during over 1-year follow-up.

As we know, muscular changes during maxillofacial surgery. In mandibular angle ostectomy, due to the dissection of the masseter muscle insertion site and the removal of the mandible angel area, the masseter muscle contracts upward, showing decreasing volume (20). In orthognathic surgery, bone geometry changes muscles’ position and shape, thus changing biomechanical conditions (21). Unlike the procedure described above for bone movement at one time, our results showed that distraction increased muscle volume and muscle length in patients with bone immaturity. Consistently, existing animal experiments demonstrated increasing volume and histochemistry regeneration of masseter muscle in temporary response to distraction (6). Theoretically, the mechanism of distraction gives movement to the mandible. It is reasonable to think that distraction can stretch the connected muscles, increasing their tension and that the muscles can transmit corresponding forces, increasing thickness reactivity (22). However, we found no significant difference in masseter width between pre - and postoperatively, in part due to large individual variations. At the second follow-up, the volume and length of the masseter were reduced compared with the first time but still higher than before the operation, indicating that the stability of MDO in the reconstruction of the masseter muscle was not ideal. It is suggested that clinical masseter long-term treatment is still inadequate.

Additionally, masseter volume is an important index for predicting masseter function (23). Preoperative results showed lower muscle volume on the affected side and correlated with M grades in OMENS + classification, consistent with previous studies (24). This emphasizes the value of evaluating masseter muscle morphology in diagnosing and treating HFM. It is known that masticatory biomechanics and masseter fiber type has plasticity (25). Hypothetically, functional exercises such as taking hard food and exercise therapy may facilitate the regeneration of masseter before bone maturity.

This study has limitations: the lack of functional data could not explain how MDO further affects masseter muscle function, and the change in resting equilibrium after distraction will have long-term effects that need further observation. Expansion of the sample size is still required to investigate how age-related masseter muscle affects the reconstruction and recurrence of the affected mandible. Conclusively, the results suggest that MDO surgery alone could improve muscle volume in the short term. Soft tissue reconstruction with functional therapy is still required for comprehensive long-term treatment of HFM.

## Data Availability

The raw data supporting the conclusions of this article will be made available by the authors, without undue reservation.
